# Shipborne oceanic high-spectral-resolution lidar for accurate estimation of seawater depth-resolved optical properties

**DOI:** 10.1038/s41377-022-00951-0

**Published:** 2022-09-02

**Authors:** Yudi Zhou, Yang Chen, Hongkai Zhao, Cédric Jamet, Davide Dionisi, Malik Chami, Paolo Di Girolamo, James H. Churnside, Aleksey Malinka, Huade Zhao, Dajun Qiu, Tingwei Cui, Qun Liu, Yatong Chen, Sornsiri Phongphattarawat, Nanchao Wang, Sijie Chen, Peng Chen, Ziwei Yao, Chengfeng Le, Yuting Tao, Peituo Xu, Xiaobin Wang, Binyu Wang, Feitong Chen, Chuang Ye, Kai Zhang, Chong Liu, Dong Liu

**Affiliations:** 1 Ningbo Research Institute, State Key Laboratory of Modern Optical Instrumentation, College of Optical Science and Engineering, Zhejiang University, Hangzhou, 310027 China; 2 Intelligent Optics & Photonics Research Center, Jiaxing Key Laboratory of Photonic Sensing & Intelligent Imaging, Jiaxing Research Institute, Zhejiang University, Jiaxing, 314000 China; 3 Univ. Littoral Côte d’Opale, CNRS, Univ. Lille, IRD, UMR 8187 - LOG - Laboratoire d’Océanologie et de Géosciences, F-62930 Wimereux, France; 4 Institute of Marine Sciences (ISMAR), Italian National Research Council (CNR), Rome - Tor Vergata, 00133 Italy; 5 Sorbonne Université, CNRS, LATMOS, 96 Boulevard de l’Observatoire, 06304 Nice Cedex, France; 6 Scuola di Ingegneria, Università della Basilicata, Viale Ateneo Lucano 10, I-85100 Potenza, Italy; 7 Cooperative Institute for Research in Environmental Sciences, University of Colorado Boulder and NOAA Chemical Sciences Laboratory, 325 Broadway, Boulder, CO 80305 USA; 8 Institute of Physics, National Academy of Sciences of Belarus, Pr. Nezavisimosti 68-2, Minsk, 220072 Belarus; 9 Key Laboratory for Ecological Environment in Coastal Areas (State Oceanic Administration), National Marine Environmental Monitoring Center, Dalian, 116023 China; 10 CAS Key Laboratory of Tropical Marine Bio-Resources and Ecology, South China Sea Institute of Oceanology, Chinese Academy of Sciences, Guangzhou, 510301 China; 11 School of Atmospheric Sciences and Guangdong Province Key Laboratory for Climate Change and Natural Disaster Studies, Sun Yat-sen University, Zhuhai, 519000 China; 12 Faculty of Technology and Environment, Prince of Songkla University, Phuket, 83120 Thailand; 13 Second Institute of Oceanography, Ministry of Natural Resources, Hangzhou, 310012 China; 14 Ocean College, Zhejiang University, Zhoushan, 316021 China; 15 Donghai Laboratory, Zhoushan, 316021 China

**Keywords:** Optical techniques, Applied optics

## Abstract

Lidar techniques present a distinctive ability to resolve vertical structure of optical properties within the upper water column at both day- and night-time. However, accuracy challenges remain for existing lidar instruments due to the ill-posed nature of elastic backscatter lidar retrievals and multiple scattering. Here we demonstrate the high performance of, to the best of our knowledge, the first shipborne oceanic high-spectral-resolution lidar (HSRL) and illustrate a multiple scattering correction algorithm to rigorously address the above challenges in estimating the depth-resolved diffuse attenuation coefficient *K*_d_ and the particulate backscattering coefficient *b*_bp_ at 532 nm. HSRL data were collected during day- and night-time within the coastal areas of East China Sea and South China Sea, which are connected by the Taiwan Strait. Results include vertical profiles from open ocean waters to moderate turbid waters and first lidar continuous observation of diel vertical distribution of thin layers at a fixed station. The root-mean-square relative differences between the HSRL and coincident in situ measurements are 5.6% and 9.1% for *K*_d_ and *b*_bp_, respectively, corresponding to an improvement of 2.7–13.5 and 4.9–44.1 times, respectively, with respect to elastic backscatter lidar methods. Shipborne oceanic HSRLs with high performance are expected to be of paramount importance for the construction of 3D map of ocean ecosystem.

## Abstract (web summary)

Shipborne oceanic high-spectral-resolution lidar for accurate estimation of seawater depth-resolved optical properties

## Graphical abstract

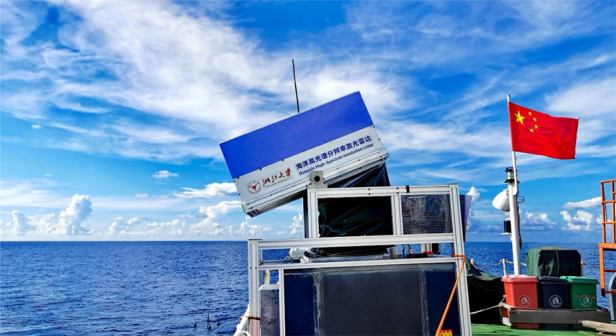

## Introduction

Lidar techniques present distinctive abilities in ocean remote sensing, providing continuous vertical information of optical properties within the upper water column during both day- and night-time^1–3^. These techniques allow improving our understanding of marine ecosystems and biogeochemistry including the diel vertical migration of marine species^2^, carbon cycles^4^, annual cycles of polar phytoplankton abundance^1^, phytoplankton layers^5,6^ and Antarctic spring ice-edge blooms^7^. In addition to this, future perspectives for the lidar look bright because many scientific studies that could be effectively carried out, e.g., phytoplankton^8,9^, carbon cycle^10^, mesoscale eddies^11^ and polar regions^12^, lack continuous, diel, depth-resolved data. However, the ill-posed nature of elastic backscatter lidar retrievals and multiple scattering effects could limit the accuracy of existing lidar techniques, which harpers our investigation of marine ecosystems and biogeochemistry using lidar^3,13^. A major limitation of the elastic backscatter lidar is that it needs to infer two unknowns, attenuation and backscatter, from a single measurement, leading to an ill-posed problem^3^. Besides, the laser propagation in seawater is accompanied by strong, nonnegligible multiple scattering effects, determining substantial difficulties in the retrieval, e.g., reliable products of the attenuation^14^, size and shape^15,16^ of ocean particles.

A variety of efforts have been made to solve the ill-posed problem of the lidar equation. Initially, various algorithms were proposed without changing the mechanism of elastic backscatter lidar, including slope method^17^, Fernald method^18^ and perturbation method^17^ etc. Nevertheless, errors are still inevitable for each method has its own set of imperfect assumptions^3^. An important leap in retrieval accuracy has been achieved with the high-spectral-resolution lidar (HSRL) technique, which can independently measure backscattering and attenuation by separating the particulate and molecular backscatters in wavelength distribution. This technique has been used for decades in aerosols and clouds measurements based on Cabannes-Brillouin scattering from air molecules with broadening of ~3 GHz^19–22^, and was recently developed for aircraft deployment in ocean detections^23,24^ using the backscatter from water molecules shifted to both sides by ~7–8 GHz at 532 nm^25–27^. Despite high efficiency of airborne HSRL, its simultaneous measurements with in situ methods are difficult. Therefore, at scenes of multi-parameter observations, e.g., detections of temperature, salinity, current and phytoplankton when investigating interactions between physical ocean processes and ocean ecosystems, it would be beneficial to develop shipborne HSRL that can endure the harsh conditions on board and work with other shipborne equipment by providing in-flow continuous measurements over the depth.

Owing to the complexity of multiple scattering, several methods have been proposed to improve the retrieval accuracy based on the elaboration of effective theoretical models to analyze oceanic HSRL signals, mainly ranging from Monte Carlo (MC) simulations^28,29^ to the simplified radiation transfer theory^30,31^. Typically, Gordon^29^ suggested that signal attenuation is identified as the beam attenuation coefficient if the field of view (FOV) was small enough, while a large enough FOV corresponds to the diffuse attenuation coefficient. However, it is difficult to evaluate if the FOV is not large or small enough. Besides, it does not consider that the attenuation changes throughout the whole detection depth under multiple scattering. Walker and McLean^30^ suggested that the attenuation coefficient is approximately equal to the absorption coefficient in shallow water and the diffuse attenuation coefficient in deep water, which avoided the FOV influence but still did not solve the problem of depth. Therefore, large errors could be introduced using these experiential conclusions. It would be extremely urgent to develop an algorithm to remove the multiple scattering effect on the attenuation considering the impacts of the FOV and depth.

We developed, to the best of our knowledge, the first shipborne oceanic HSRL and a multiple scattering correction (MSC) algorithm for rigorously addressing the above-mentioned challenges, the ill-posed nature of elastic backscatter lidar retrievals and multiple scattering. Underway continuous HSRL measurement are reported together with the first diel measurement at a fixed station, that were collected in the coastal areas of East China Sea (ECS) and South China Sea (SCS), which are connected by the Taiwan Strait. Measurements were carried out during both day- and night-time as a part of the 2020 Autumn Joint ECS&SCS cruise. In addition, comparisons between retrievals using HSRL, elastic backscatter lidar and in situ measurements were carried out.

## Results

### Shipborne oceanic HSRL

**Fig. 1:**
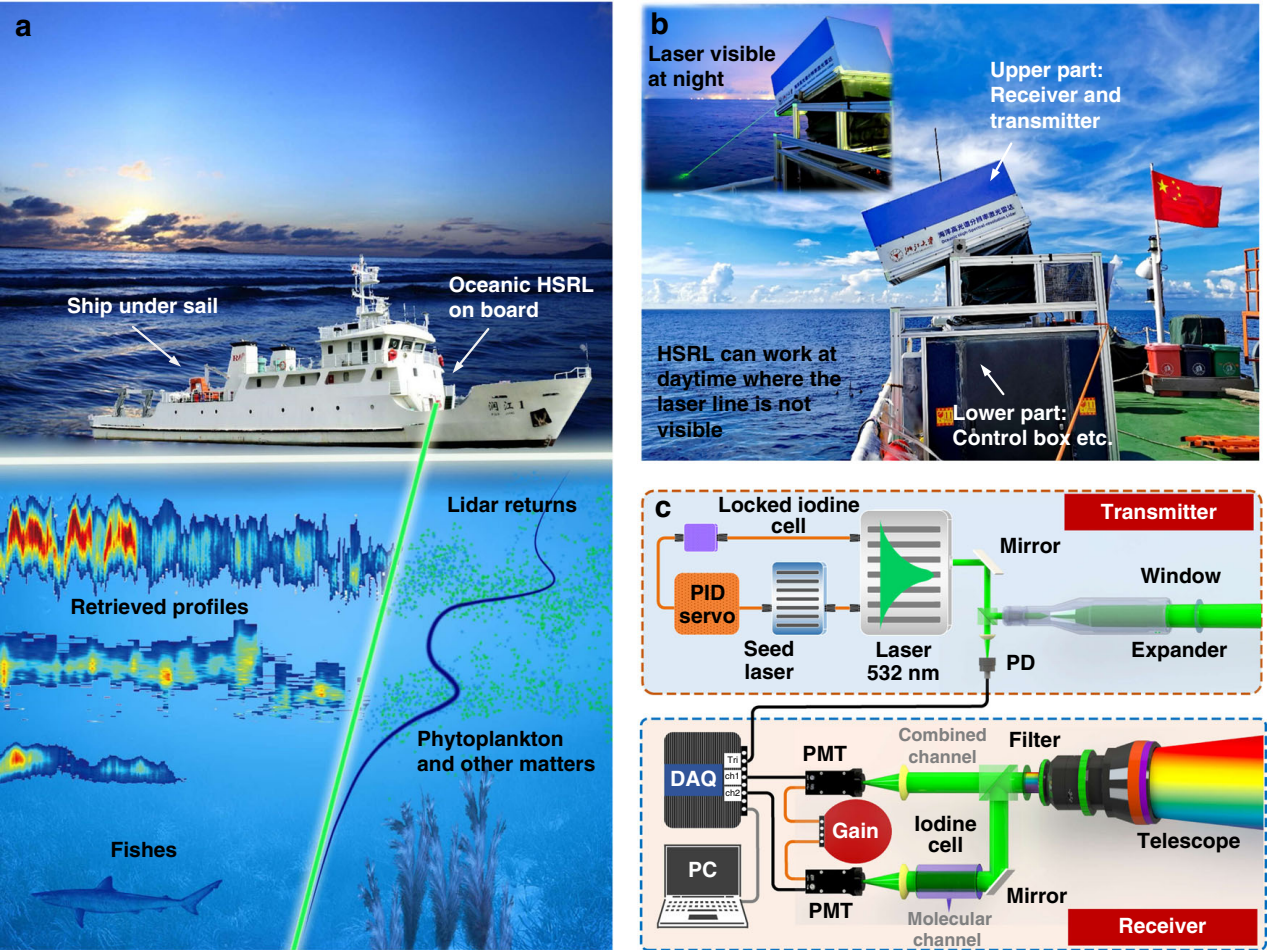
Principle of the shipborne oceanic HSRL. **a** Concept of the shipborne HSRL profiling seawater optical properties. **b** HSRL can work at both day- and night-time. **c** Schematic diagram of the HSRL system with the iodine absorption cell discriminator

The operational principle of the shipborne oceanic HSRL is shown in Fig. 1. It transmits a laser pulse into the seawater and collects the backscattered echoes, known as the lidar signal, which contains depth-resolved seawater information, as shown in Fig. 1a. The attenuation and backscatter of laser radiations, and consequently the lidar signals, are sensitive to all optically active constituents, e.g., pure seawater, phytoplankton, colored-dissolved-organic matter (CDOM) and non-algal particles, etc. For example, a phytoplankton layer may generate a peak in the elastic backscatter signal, while CDOM only contributes to the attenuation. The HSRL system is divided into two major subsystems (Fig. 1b). The upper subsystem mainly consists of the laser head and the receiver, which can rotate from the horizontal position to nadir. Thus, a same lidar geometrical factor can be estimated after calibration in the horizontal position of atmosphere. The lower subsystem consists of computer, control boxes, a water-cooling system, a power supply and an air conditioner etc., which support the operation of the upper part. The system is integrally sealed to avoid the erosion from the sea salt and sea foam. Due to the strong background light, the laser light is not visible at day-time but it is clearly visible at night-time (up-left of Fig. 1b). The HSRL incident angle is ~60 degree during underway observation to avoid the ship spray and ~40 degree at the fixed station. The laser polarization direction is perpendicular to the light incident plane to ensure a small air-sea Fresnel reflectance at above the incidence angles^32^.

The schematic diagram of the shipborne oceanic HSRL is illustrated in Fig. 1c and the key technical specifications of the HSRL are listed in Supplementary Table S1. An ultra-narrow (picometer) spectral discrimination is achieved through a series of transmitting and receiving techniques. Specifically, a diode-pumped, Q-switched, injection-seeded, frequency-doubled Nd-YAG laser at 532 nm is used, with a pulse energy of 10 mJ and a repetition frequency of 10 Hz. Through a proportional-integral-derivative (PID) servo loop, the frequency of the laser is locked to the absorption line of an iodine cell. The iodine stabilized laser is then transmitted into the water via an expander and a window. A photoelectric detector (PD) is used for triggering. When the laser hits the seawater, various optical interactions occur, including Rayleigh, Brillouin, Raman scattering from water molecules, particulate scattering and fluorescence from chlorophyll-a and CDOM, etc. The backscattered signal components are collected by a telescope with a diameter of 50.8 mm and an FOV of 200 mrad. The use of an interference filter centered at 532 nm with a bandwidth of 3 nm allows filtering out Raman scattering and fluorescence with shifted wavelengths, as well as most of background radiation. Then, transmitted signal components are split into two beams, conveyed to the combined and molecular channels, respectively. The combined channel collects all the components, while the molecular channel exploits the iodine cell ultra-narrow spectral discrimination (See methods for detail) to reject both the particulate and Rayleigh signals and transmits Brillouin signal. Photomultiplier tubes (PMT) and a high-speed data acquisition card with a sampling frequency of 400 MHz, equivalent to the distance resolution of 0.28 m in the water, are used to detect and sample the lidar signals.

### Data processing

**Fig. 2:**
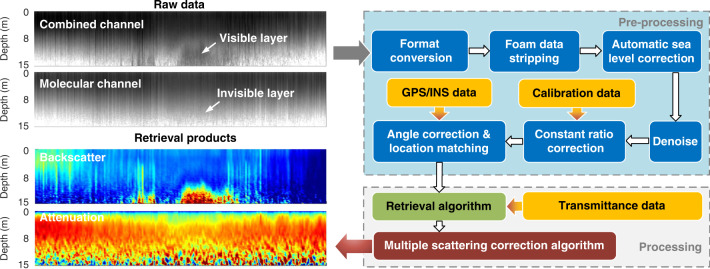
Flow chart of HSRL data processing. Steps from raw data to retrieval products

Flow chart of data processing from the raw data to retrieval products is illustrated in Fig. 2. The lidar signals of the combined and molecular channels are regarded as the raw data, as shown in up-left of Fig. 2. The scattering layer can be seen in the combined channel, while it disappears in the molecular channel as the particulate scattering is rejected by the discriminator. The preprocessing flowchart of the HSRL data are shown in up-right of Fig. 2. For a large FOV, the geometrical factor reaches unit after several meters in the atmosphere and will not influence signals in the water so it is not considered in the data preprocessing. The raw data are converted into standard format with time, location and system status. Then, the automatic removal of the sea foam (Supplementary Fig. S1) and identification of the sea level are carried out to eliminate the impact of waves and ship fluctuation on the signal. For the denoising algorithm, the influence of the background and random noises on the signal is filtered out^28^ (Supplementary Fig. S2). Then, the data of 0–3 m are removed to eliminate surface effects (e.g., bubbles) and the data with low SNR are removed, which are defined as the dynamic range of ~3–3.5 orders of magnitude considering the influence of the noise and tailing of system response. Furthermore, the detector gain ratio and channel efficiency ratio are calibrated for the constant ratio corrections. The interference of ship shaking and navigation location matching are considered using Global Position System (GPS) and Inertial Navigation System (INS) data. The pre-processing signals in combined and molecular channels are finally obtained as inputs of the retrieval algorithm. The processing flowchart of the HSRL data are shown in lower-right of Fig. 2. The retrieval algorithm outputs diffuse attenuation coefficient *K*_d_, particulate backscatter coefficient *b*_bp_ and the lidar ratio *R*. Then, an MSC algorithm is proposed to revise *K*_d_ as well as *R* (see details in Methods). The retrieval products of *K*_d_ and *b*_bp_ are shown in lower-left of Fig. 2

### Underway measurements

**Fig. 3:**
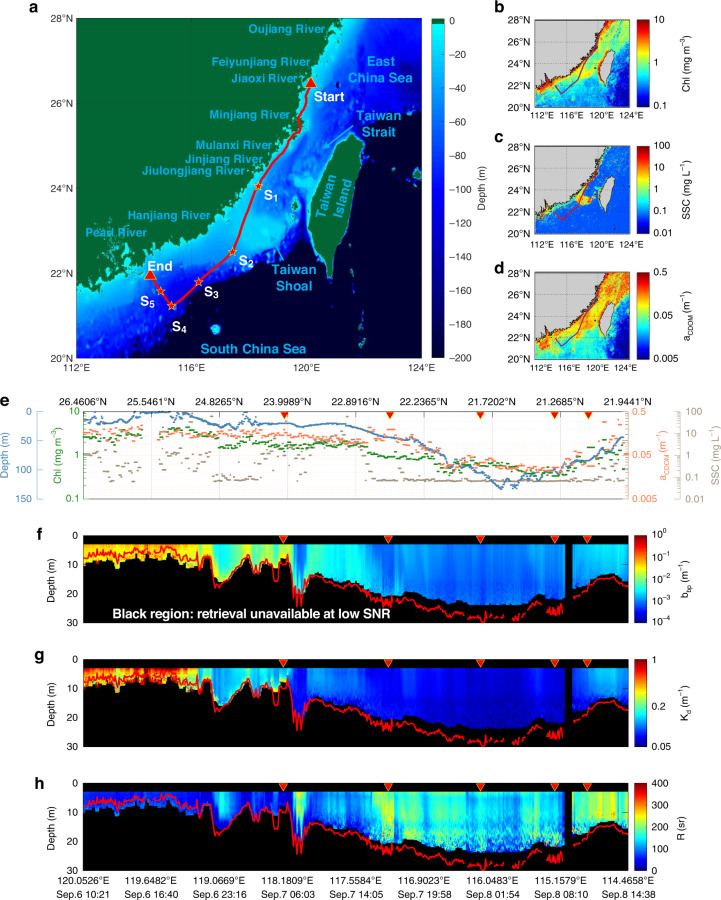
Continuous underway HSRL measurements (~800 km). Plots of **a** water depth from ETOPO1, **b** Chl, **c** SSC, **d** *a*_CDOM_ from HY-1C (Methods) with the ship track represented with the red lines. Discrete in situ measurements were obtained at stations S_1_-S_5_ (Methods). **e** Values along the ship track in Fig. 3a–d. Profiles of **f** *b*_bp_, **g** *K*_d_, **h** *R* retrieved by HSRL with 3 optical depths in red lines

During the Autumn 2020 Joint ECS and SCS cruise with the *R/V Runjiang No.1*, continuous underway HSRL measurements (~800 km) were carried out from Sep. 6 to Sep. 8 with the ship track and study region shown in Fig. 3a. The digital topographic data is from the ETOPO1 Global Relief Model (Methods). Discrete in situ optical properties, temperature and salinity were obtained at stations S_1_-S_5_ (Methods). The spatial variability of the water column bio-optical properties, namely the chlorophyll-a concentration (Chl), the suspended sediment concentration (SSC) and the colored dissolved organic matter absorption at 440 nm (*a*_CDOM_) is illustrated using the L2B products of the China ocean color satellite HY-1C, as shown in Fig. 3b–d, respectively (Methods). Then, the water depth versus Chl, SSC and *a*_CDOM_ along the ship track (red lines) of Fig. 3a–d are illustrated in Fig. 3e.

The profiles of *b*_bp_, *K*_d_ and *R* by HSRL are plotted in Fig. 3f–h. The red lines in Fig. 3f–h are 3 optical depths that are defined as the product of the depth and the attenuation coefficient, which is used for the assessment of lidar detection depth^3^. The black regions in Fig. 3f–h are not considered due to the low signal-to-noise ratio (SNR). In the region close to the Zhejiang and the Fujian Provinces, namely the north section from Start (start of measurement session) to S_1_, values of Chl, SSC and *a*_CDOM_ (Fig. 3e) are high^33,34^ since most of the sediments transported by rivers are trapped in estuaries or deposited on adjacent continental shelves of ECS^35^. *b*_bp_ and *K*_d_ values of ~0.1 m^−1^ and ~0.5 m^−1^ lead to a detection depth generally smaller than 10 m as the SNR decreases rapidly. For the Taiwan Strait area (the south section from Start to S_1_), Chl and *a*_CDOM_ values (Fig. 3e) are similar to those in the north section, while SSC values change dramatically. *b*_bp_ and *K*_d_ values are lower and the detection depth can reach 10–15 m. In the south part of Taiwan Strait (from S_1_ to S_2_), SSC values (Fig. 3e) of the Taiwan Shoal are much higher than values in the surrounding areas that presumably include open ocean water^33^. Similarly, values of *b*_bp_ and *K*_d_ are ~0.0045 m^−1^ and ~0.1 m^−1^, respectively, in the Taiwan Shoal, but decrease to ~0.001 m^−1^ and ~0.08 m^−1^, respectively, in the surrounding areas. For the northern SCS area, which is close to Guangdong Province (from S_2_ to S_4_), values of Chl, SSC and *a*_CDOM_ (Fig. 3e) are much lower and SSC is almost close to the minimum detection limit because generally SCS is much clearer than ECS^36^. Similarly, values of *b*_bp_ and *K*_d_ decrease to ~0.001 m^−1^ and ~0.07 m^−1^, respectively. From S_4_ to End (end of measurement session), gradually approaching the shoreside, values of Chl and *a*_CDOM_ gradually increase, while SSC values remain low. Values of *b*_bp_ and *K*_d_ are ~0.002 m^−1^ and ~0.11 m^−1^, respectively. Generally, trends in *b*_bp_ and *K*_d_ from HSRL measurements seem to agree with those observed for the Chl, SSC and *a*_CDOM_ products derived from HY-1C.

The lidar attenuation to backscatter ratio *R* from HSRL is an important index for the characterization of water composition (Fig. 3h). Churnside et al.^37^ reported lidar ratio values close to 100 for values of Chl lower than 3 mg m^−3^ for Case 1 water, where phytoplankton abundance is high compared to nonbiogenic particles and absorption by Chl and related pigments represents a major contribution in the total absorption coefficient^38^. Obviously, the experimental water belongs to Case 2 water that are “everything else except Case 1”^38^ since Chl is not the only dominant factor as shown in Fig. 3e. Chl, SSC and CDOM contribute to the light absorption while only Chl and SSC contribute to light backscattering. Generally, *R* values in Fig. 3h are lower in the transect ranging from Start to S_2_ than from S_2_ to End because high SSC concentrations determine the high backscatter values found in the former transect, while SSC values decrease to nearly zero and Chl and CDOM dominate in the latter transect. It is to be specified that almost constant SSC values of ~1 mg L^−1^ from Start to S_2_ produce different values of *R*, e.g., ~50 sr from Start to S_1_ but ~150 sr from S_1_ to S_2_ perhaps due to the monthly average of SSC. Interestingly, between S_1_ and S_2_, values of *R* of ~80 sr in the Taiwan Shoal are much lower than its surrounding areas (~220 sr) centered at (118.05°E, 23.82°N) and S_2_ (117.33°E, 22.50°N). For S_2_, high *a*_CDOM_, low Chl and very low SSC values could explain high *R* values. Around (118.05°E, 23.82°N), values of SSC highly fluctuate, but Chl and *a*_CDOM_ values are similar to those found in the Taiwan Shoal so it is possible that the sailing route crossed the front between the ECS Coastal Current and Taiwan Warm Current preventing the high SSC water from being transported to the shelf^33^. In addition, *R* values from S_5_ to End are larger than those found in the Taiwan Shoal between S_2_ and S_3_ while their *K*_d_ are similar, perhaps due to the SSC variability from ~3 mg L^−1^ to 0.1 mg L^−1^. *R* values can also be different at different vertical levels. For example, *R* values observed for the scattering layer moving from a depth of 5 m to a depth of 10 m around (24.8265° N, 119.0669° E) are much smaller than those found outside such layer, as shown in Supplementary Fig. S3. A possible motivation for this variability is that the layer is close to the water bottom and is characterized by high SSC, which in turn can reduce *R* values. Overall, for most of the data collected during the cruise, the water column composition, especially the values of SSC, is highly correlated to *R* values.

### Diel measurement

**Fig. 4:**
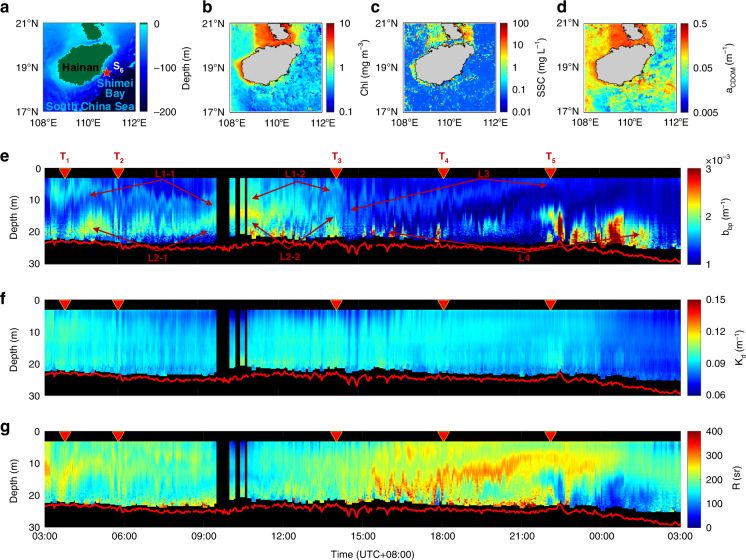
A diel continuous measurement for a fixed station. Plots of **a** water depth from ETOPO1, **b** Chl, **c** SSC, **d** *a*_CDOM_ from HY-1C (Methods) with the ship track in red lines. Profiles of **e** *b*_bp_, **f** *K*_d_, **g** *R* retrieved by HSRL with 3 optical depths in red lines. Discrete in situ data were collected at different time T_1_-T_5_ (Methods)

During the Autumn 2020 Joint ECS&SCS cruise, a diel continuous measurement was carried out on Sep. 14, 2020 at a fixed station S_6_, which is close to the Shimei Bay of the SCS, as shown in Fig. 4a–d. The water depth is 48 m and the Chl, SSC and *a*_CDOM_ values derived from HY-1C at this station were 0.54 mg m^−3^, 0.07 mg L^−1^ and 0.04 m^−1^, respectively (Methods). The sunrise and sunset time were 06:25 and 18:42 (UTC + 08:00), respectively. Note that the UTC + 08:00 is adopted in the following text unless otherwise stated. Discrete in situ measurement of the optical properties, temperature and salinity were collected at different time (T_1_–T_5_) (Methods).

The *b*_bp_, *K*_d_ and *R* values retrieved from HSRL data are shown in Fig. 4e, f, respectively, where the first lidar diel continuous observation of scattering layers for a given fixed station is presented. Since *b*_bp_ is sensitive to the suspended particulate matter, it is not surprising to find scattering layers named as L1-1, L1-2, L2-1, L2-2, L3 and L4 in Fig. 4e throughout the whole day. L1-1 is about 2 m thick and located at the depth of ~5 m at 03:00. However, the depth gradually increases to ~10 m at 09:00. L1-2 layer, that is most probably the continuation of L1-1, shows a depth decrease from ~10 m at 9:00 to ~5 m at 14:00. L2-1 and its possible continuation L2-2 fluctuated around a depth of 15 m from 03:00 to 14:00. Apparently, L1-2 and L2-2 gradually merged into L3 after 14:00 for which the depth gradually decreased up to ~5 m from 15:00 to 00:00. L4 was intermittent from 10:00 to 24:00 as the depth then was beyond the detection range of the HSRL. From 18:00 to 22:00, its depth became shallower and the background noise became lower so that it could be detected continuously. In addition, it is interesting that the wild fluctuations of depths of all layers in a short time are simultaneous. The appearance of layers is probably caused by the internal waves. As shown in Supplementary Fig. S4, Sentinel-1A Synthetic Aperture Radar (SAR) quick-look image of internal waves in the Shimei Bay is taken at the same day as the lidar observation (10:46:16 UTC on Sep. 14, 2020). Although internal wave edges are not very clear, it presents a similar pattern as demonstrated by Churnside and Ostrovsky^39^. Notably, *R* values observed within the layers are smaller than those of the surrounding areas (Fig. 4g), which can be also observed around T_5_ (see details in Supplementary Fig. S5), thus allowing to infer the change of water column properties.

In Fig. 4e and Supplementary Fig. S6a, the increase of *b*_bp_ appeared in the time intervals 09:00–15:00 and 21:00–00:00. The seawater temperature and salinity, measured every 2 h, were subtracted by their mean values at each depth to obtain their anomalies, as shown in Supplementary Fig. S6b, c. It is interesting to find a decreased temperature and increased salinity between 09:00–15:00 and 21:00–00:00, which might be caused by upwelling that brought the deep cold water with high salinity and nutrient to the surface. Considering sunlight contribution, it is possible that an increase of both nutrient and light led to the phytoplankton rising between 09:00–15:00, while the increase of *b*_bp_ between 21:00–00:00 might be attributed to the upwelling of phytoplankton and sediment close to the bottom.

### Consistency check

**Fig. 5:**
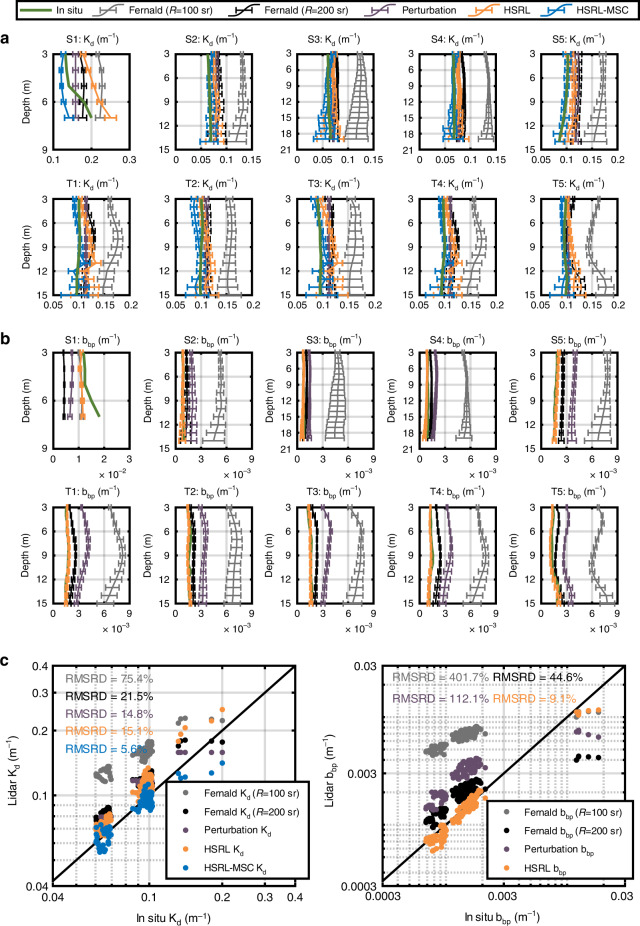
The consistency check of HSRL retrievals. **a** Comparisons of *K*_d_. **b** Comparisons of *b*_bp_. The orange represents outputs of HSRL retrieval algorithm and blue refers to *K*_d_ of HSRL retrieval and MSC algorithm in Fig. 2. Fernald method based on lidar ratios of 100 sr (gray) and 200 sr (black) and perturbation method (purple) are used for the elastic backscatter lidar. All lidar results are validated by in situ measurements (green) in **c** statistical analysis of *K*_d_ and *b*_bp_

As shown in Fig. 5, *K*_d_ and *b*_bp_ values from HSRL are validated using in situ measurements (green) and compared with several methods applied to the elastic backscatter lidar signals from the HSRL (Methods). The orange represents outputs of HSRL retrieval algorithm and blue refers to *K*_d_ of HSRL-MSC algorithm in Fig. 2. The methods applied to the elastic backscatter lidar signals include the Fernald method^18^ based on different lidar ratios of 100 sr (gray) and 200 sr (black) and the perturbation method^5^ (purple). The data at S_1_-S_5_ in Fig. 3 and at T_1_-T_5_ in Fig. 4 are adopted.

As shown in Fig. 5a, it reveals that *K*_d_ values from the Fernald method (*R* = 100 sr adopted for Case I water^37^) deviate furthest from the in situ results due to the inappropriate *R* value^18^, as shown in Supplementary Fig. S7. After changing *R* to 200 sr, *K*_d_ of Fernald method decreased and approached to the in situ results. Although *R* is still smaller than the in situ results, as shown in Supplementary Fig. S7, the consistency seems good due to the algorithm convergence. The perturbation method only outputs a constant *K*_d_ value along the water column but is close to in situ data probably because of the small influence of inhomogeneous backscatter on the attenuation. As for HSRL, the vertical distribution of *K*_d_ can be obtained independently without a priori assumptions of the lidar ratio. However, the derived *K*_d_ value is slightly larger than the in situ measurements because of the strong influence of multiple scattering. Then, *K*_d_ agrees better with the in situ values after adopting MSC algorithm.

Furthermore, the higher accuracy of HSRL over elastic backscatter lidar can be well supported by the *b*_bp_ results, as shown in Fig. 5b. Generally, the degrees of deviation from the in situ values follow the scheme “Fernald (100) > perturbation > Fernald (200) > HSRL” although it is not similar for S_1_ perhaps due to small in situ lidar ratio. In Supplementary Fig. S7, perturbation method obtains a similar *R* of 100 with Fernald (100) because they are both based on the bio-optical method of Case 1 water. However, the effects of unreasonable *R* on the accuracy of Fernald method seem larger than that of perturbation method. If *R* values are changed to 200 sr, the accuracy of Fernald method is improved.

Then, the results are analyzed in Fig. 5c. The root mean square relative difference (RMSRD) is utilized here for reflecting the HSRL performance realistically, that is,

1 $${{{\mathrm{RMSRD(\% ) = 100}}}}\sqrt {\frac{{\mathop {\sum}\nolimits_{i = 1}^N {\left( {x_i/\widetilde x_i - 1} \right)^2} }}{N}}$$

where *N* is the total number of sampling points, *x*_*i*_ is the lidar-measured value and $\widetilde x_i$ is the in situ measured value. As shown in Fig. 5c, it shows that the RMSRD of *K*_d_ and *b*_bp_ ranges from 15.1–75.4% and 44.6–401.7% for the methods of elastic backscatter lidar, respectively. The RMSRD of *K*_d_ between the HSRL and in situ measurements is 15.1%, and that of *b*_bp_ is 9.1%. Furthermore, the MSC algorithm reduces the RMSRD of *K*_d_ by a factor of 2.7, typically from 15.1% to 5.6%. Overall, the HSRL-MSC results remarkably improve the accuracy by factors of 2.7–13.5 and 4.9–44.1 for *K*_d_ and *b*_bp_, respectively, relatively to the elastic backscatter lidar retrievals. The strong correlations (*r*^2^ = 0.86 for log_10_(*K*_d_) and *r*^2^ = 0.94 for log_10_(*b*_bp_)) were also observed between in situ and HSRL-MSC measurements. In general, HSRL and HSRL-MSC demonstrate more accurate estimations of *K*_d_ and *b*_bp_ than elastic backscatter lidar methods.

## Discussion

In this paper, we developed, to the best of our knowledge, the first shipborne oceanic HSRL for which none a priori assumptions of the lidar ratio are required. It is achieved by using an ultra-narrow spectral discrimination in the picometer order through a series of techniques including single longitudinal mode laser, iodine absorption cell with accurate controlling of temperature and frequency locking part. An MSC algorithm for rigorously addressing the multiple scattering is proposed to further promote the retrieval accuracy. The HSRL-MSC RMSRDs are 5.6% for *K*_d_ and 9.1% for *b*_bp_ during the 2020 Autumn Joint ECS&SCS cruise, which remarkably improves the accuracy by factors of 2.7–13.5 and 4.9–44.1 for *K*_d_ and *b*_bp_, respectively, relatively to the elastic backscatter lidar methods. The accuracy are a little better than those of 17.2% for *K*_d_ and 27.1% for *b*_bp_ from the NASA airborne HSRL system^23^, which might be related to several factors, although the retrieval accuracy varies amongst study areas. There are often spatial and temporal gaps between the lidar and in situ measurements, which may cause different measured water^23,40^. Fortunately, the gaps can be almost ignored in this paper since the lidar and in situ method both worked on the ship. Furthermore, the utilization of MCS algorithm could be another reason that promotes the accuracy of *K*_d_. Note that the algorithm can also be adopted at other lidar systems after changing the system parameters.

Although airborne and spaceborne lidar can cover regional or global ocean^1,2,4–6,16,24^, shipborne lidar can illuminate our understanding of the underlying biological/physical/chemical processes in ocean by combining lidar and other shipborne measurements^8,24,41–43^. For example, when any anomaly is detected, the in situ measurements can be easily deployed to validate and investigate the HSRL results (Supplementary Fig. S6). Moreover, shipborne lidar has potential to penetrate deeper than airborne or space-borne lidars because of less atmosphere influence and closer distance to water surface. Also, the shipborne oceanic HSRL can provide continuous measurements of the depth-resolved bio-optical properties, which is more difficult to get from in situ measurements on the ship, e.g., WETLabs acs used in this paper. Considering the huge advantages of accuracy, simultaneous measurements with in situ methods and spatial-temporal sampling, the shipborne HSRL proposed in this study could be of primary importance for the following issues: 1) to improve the understanding of ocean biological, physical and biogeochemical processes by resolving the vertical structure within the upper water column; and 2) to validate the existing and future spaceborne lidar missions dedicated to the ocean.

Thin layers that are often related to ocean biological and physical process attract wide interests^8,43^. However, it is difficult to find layers based on in situ measurements due to the complex sampling steps and limited sampling stations. HSRL technique increases the likelihood to detect layers through continuous observation, allowing direct quantification of thin layer characteristics and dynamics. Also, the shipborne HSRL could be conveniently operated together with other sampling technologies to recognize the layer compositions and understand the associated environmental conditions^44^. Except thin layers, the shipborne HSRL is able to characterize the vertical heterogeneity of other matters in the ocean, which are expected to contribute to many scientific studies, such as carbon cycle^4^ and eddies^11^.

The lidar ratio *R* of HSRL can be a proxy of the Chl:*b*_bp_ ratio because Chl is directly related to *K*_d_ in Case 1 waters. Such a ratio has been proved useful for tracking changes in phytoplankton community composition^24,45^. Low values of Chl:*b*_bp_ ratio are associated with pico- and nano-phytoplankton while high values are associated with diatom dominated phytoplankton communities^45^. Schulien et al.^24^ showed that changes in airborne HSRL measurements coincided with a shift in phytoplankton community composition across an anticyclonic eddy in the North Atlantic. In addition, the Chl:*b*_bp_ ratio can reflect the photoacclimation state of the phytoplankton cell^9^.

The powerful performance of shipborne HSRL is of great interest for the validation of the current and future spaceborne lidar missions for ocean observation. Recently, spaceborne lidars such as Cloud-Aerosol Lidar and Orthogonal Polarization (CALIOP) greatly promoted our knowledge about phytoplankton in polar regions^1^ and daily vertical migrations of ocean animals^2^. Besides, the additional information on the size and the shape of ocean particles potentially can be provided from CALIOP^16^. Another spaceborne lidar designed for ice, cloud and land elevation measurements, Advanced Topographic Laser Altimeter System (ATLAS), was able to provide information on Antarctic spring ice-edge blooms^7^. Future spaceborne lidar missions dedicated to ocean will further improve our understanding of ocean ecosystem functioning^3^. However, there are only a few studies that validated such an approach^4,40^. Therefore, it will be useful and convenient for shipborne HSRL to significantly contribute to supporting the validation of the spaceborne lidar.

Despite development of shipborne oceanic HSRL, the potential of lidar has not been fully realized. In the future, it is expected to develop multi-wavelength HSRL based on the existing HSRL at 532 nm^3,6^. The fielded-widen Michelson interferometric spectral filter or Fabry-Pérot interferometric filter can be utilized as optional ultra-narrow discriminators in wavelengths outside 532 nm^26^ but the contradiction between the more wavelengths and more difficult manufacturing technique of laser will limit the lidar wavelength. The multi-wavelength technique can provide more information about CDOM, Chl and bring us to the bottom of euphotic layer in the open ocean, which can strengthen our understanding of the maximum Chl layer. In addition, with the development of oceanic polarized lidar simulation technique, it is possible to interpret the depth-resolved depolarized information from multiple scattering^46^. The information of depolarization is directly related to the microphysical information of particles, like shapes, which could improve our understanding of phytoplankton species community^15,16^.

## Materials and methods

### Ultra-narrow discrimination

**Fig. 6:**
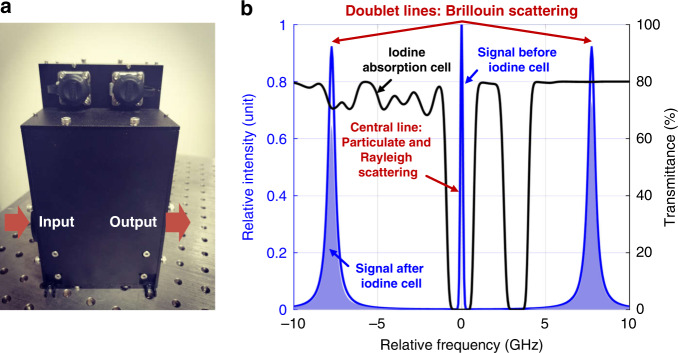
The ultra-narrow discrimination of the iodine cell. **a** The picture of the packed iodine absorption cell integrated with the temperature controller. The light enters the device from the left and leaves from the right. **b** Illustration of the spectral discrimination, where the iodine cell (black line) filters the signal (blue line) by rejecting the particulate and molecular Rayleigh signals and transmitting molecular Brillouin signal (blue shadow)

The iodine cell is exploited in the molecular channel for ultra-narrow spectral discrimination, as shown in Fig. 1c. The packed iodine absorption cell is integrated with the temperature controller (Fig. 6a). The light enters the device from the left and leaves from the right. Then the cell could reject both the particulate and Rayleigh signals centered at the laser wavelength and transmits Brillouin signal shifting to the both sides of the center with a frequency shift of about 7–8 GHz (Fig. 6b). There are several iodine absorption lines that can be exploited in the HSRL technique and here we select the 1104 line (Supplementary Fig. S8), with the central wavelength at ~532.2928 nm. The laser emission, the particulate backscatter signals and 1104 iodine line have coincident wavelengths.

### HSRL retrieval algorithm

The retrieval algorithm of HSRL has already been proposed^25,26^ but can be simplified when the molecular Brillouin transmittance *T*_B_ is large while the particulate and Rayleigh transmittance *T*_p_ and *T*_R_ is tiny, that is,

2 $$b_{{{{\mathrm{bp}}}}}(z) = 2\pi \chi \beta _{{{\mathrm{p}}}}^{\uppi}$$

where $\beta _{{{\mathrm{p}}}}^{\uppi} = \beta _{{{\mathrm{B}}}}^{\uppi}\left[ {T_{{{\mathrm{B}}}}B_{{{\mathrm{C}}}}(z)/B_{{{\mathrm{M}}}}\left( {z - 1} \right.} \right]$, *B*_C_ and *B*_M_ are the signals of combined and molecular channels, *z* is the water depth, $\beta _{{{\mathrm{B}}}}^{\uppi}$ and $\beta _{{{\mathrm{p}}}}^{\uppi}$ is the Brillouin and particulate 180° volume scattering function^38^, *χ* is the conversion factor that connects $\beta _{{{\mathrm{p}}}}^{\uppi}$ and *b*_bp_ with a selected value of 1.0^47^. *T*_B_ and *T*_p_ are calibrated and calculated by scanning iodine absorption lines. The lidar attenuation coefficient can be written as

3 $$k_{{{{\mathrm{lidar}}}}}(z) = - \frac{1}{2}\frac{{{{\mathrm{d}}}}}{{{{{\mathrm{d}}}}z\!/\!\!\cos \theta _r}}\ln \left[ {B_{{{\mathrm{M}}}}(z) \cdot \left( {\frac{{nH}}{{\cos \theta _i}} + \frac{z}{{\cos \theta _r}}} \right)^2} \right]$$

where *n* is the refractive index of the seawater, *H* is the HSRL working height above the water surface, *θ*_i_ and *θ*_r_ are the angles of incidence and refraction in the atmosphere and seawater, respectively. *k*_lidar_ is generally regarded as the diffuse attenuation coefficient *K*_d_ in a large FOV^29^.

Then, the lidar ratio *R* is defined as

4 $$R(z) = \frac{{K_{{{\mathrm{d}}}}(z) - K_{{{{\mathrm{d,w}}}}}}}{{\beta _{{{\mathrm{p}}}}^{\uppi}}}$$

where *K*_d,w_ is the diffuse attenuation coefficient of the pure seawater.

### Multiple scattering correction algorithm

There are several simulating and experimental results^14,23,30^ not totally support the approximation that *k*_lidar_ is regarded as *K*_d_ in a large FOV^29^. In fact, *k*_lidar_ changes with depth considering multiple scattering even in a homogeneous water^14,30^. Here, considering the relationship between inherent optical properties (IOPs) and *k*_lidar_^48^, we developed an MSC algorithm, which transfers *k*_lidar_ to *K*_d_ by correcting the effects of multiple scattering. The MSC algorithm is established as follows:

Step 1: The analytical model based on the quasi-single small-angle approximation^49^ is built for the simulation of molecular signals with a flat molecular backward phase function.

Step 2: Molecular signals are simulated using the analytical model under several IOPs conditions listed in Supplementary Table S2. Signals and *k*_lidar_ derived from Eq. (3) are plotted in Supplementary Figs. S9 and S10. Note that the angles of inclination are different in underway measurement and at the fixed station, which is considered in the simulation of Supplementary Figs. S9 and S10, respectively.

Step 3: It is interesting to find that the simulated *k*_lidar_ in Supplementary Figs. S9 and S10 have similar patterns, which is small near the surface but increase with the depth. Moreover, *k*_lidar_ seems to be proportional to the absorption. Therefore, a model for this pattern is proposed as

5 $$k_{{{{\mathrm{lidar}}}}} = m_1 \times \exp ( - m_2 \times z) + m_3 + a$$

where *a* is the absorption coefficient, *b*_b_ is the backscattering coefficient, *z* is the depth, *m*_1_, *m*_2_ and *m*_3_ are model parameters. As shown in Supplementary Figs. S9 and S10, it demonstrates good agreements between the simulated and modeled *k*_lidar_ with the values of *m*_1_, *m*_2_ and *m*_3_ shown in Supplementary Tables S3 and S4.

Step 4: The *m*_1_, *m*_2_ and *m*_3_ are then found to be described by functions based on the backscattering coefficient with high *R*^2^, as shown in Supplementary Figs. S11 and S12. It means that *k*_lidar_ can be described by *a* and *b*_b_ according to Eq. (5).

Step 5: Then, it is necessary to validate the reliability of Eq. (5). The IOPs in Supplementary Table S5 are used in simulation of molecular signals and *k*_lidar_ for validation. As shown in Supplementary Fig. S13, the modeled and simulated *k*_lidar_ show good consistency with *R*^2^ of ~0.98 and RMSRD of ~4%, where the definition of RMSRD is similar to Eq. (1).

Step 6: Combining Eq. (5) and the empirical relationship between *K*_d_ and IOPs proposed by Lee et al.^50^, the MSC algorithm can be written as:

6 $$K_{{{\mathrm{d}}}}\,{{{\mathrm{ = }}}}\,k_{{{{\mathrm{lidar}}}}} - m_1 \times \exp ( - m_2 \times z) - m_3 + 4.18 \times b_{{{\mathrm{b}}}} \times \left[ {1 - 0.52 \times e^{ - 10.8(k_{{{{\mathrm{lidar}}}}} - m_1 \times \exp ( - m_2 \times z) - m_3)}} \right]$$

Then, the MSC-corrected *K*_d_ can be obtained by substituting HSRL-measured *k*_lidar_ from Eq. (2) and *b*_b_ from Eq. (3) into Eq. (6). Then the lidar ratio *R* is updated with the MSC-corrected *K*_d_ using Eq. (4).

### Elastic backscatter lidar methods

Perturbation method, that follows the description of Churnside and Marchbanks^5^, and Fernald method^18^ (Supplementary Section S1) are used for retrievals of elastic backscatter lidar using the combined channel of HSRL as signals.

### In situ measurement

The in situ absorption and backscatter coefficients at 532 nm were collected and calculated by WETLabs acs and HOBILabs HS6P, from which the in situ *K*_d_ was derived according to the algorithm by Lee et al.^50^. The in situ temperature and salinity data were provided by a Sea-Bird Electronics, Inc. conductivity-temperature-depth device. See Supplementary Section S2 for detailed illustration.

### Topographic and remote sensing data

The digital topographic data from the ETOPO1 Global Relief Model published by the National Geophysical Data Center are available at http://www.ngdc.noaa.gov/mgg/global/global.html. The Chl, SSC and *a*_CDOM_ from L2B products of the China ocean color satellite HY-1C in Sep. 2020 are available at https://osdds.nsoas.org.cn, where *a*_CDOM_ are corrected by the field data^34^. The Sentinel-1A SAR data from European Space Agency are available at https://asf.alaska.edu/.

## Supplementary information

Supplementary Information final
