# Supplementary material for: Shipborne oceanic high-spectral-resolution lidar for accurate estimation of seawater depth-resolved optical properties
Source: Light Sci Appl. 2022 Sep 2;11:261. doi: 10.1038/s41377-022-00951-0 (PMC9440025; doi:10.1038/s41377-022-00951-0)
Supplement: Supplementary file 1 — Supplementary Information final [file 41377_2022_951_MOESM1_ESM.pdf]

Supplementary Information for

Shipborne oceanic high-spectral-resolution lidar for accurate estimation of seawater depth-resolved optical properties

Yudi Zhou[1,2,†], Yang Chen[1,†], Hongkai Zhao[1,†], Cédric Jamet[3], Davide Dionisi[4], Malik Chami[5], Paolo Di Girolamo[6], James H. Churnside[7], Aleksey Malinka[8], Huade Zhao[9], Dajun Qiu[10], Tingwei Cui[11], Qun Liu[1], Yatong Chen[1], Sornsiri Phongphattarawat[12], Nanchao Wang[1], Sijie Chen[1], Peng Chen[13], Ziwei Yao[9], Chengfeng Le[14], Yuting Tao[1], Peituo Xu[1], Xiaobin Wang[1], Binyu Wang[1], Feitong Chen[1], Chuang Ye[1], Kai Zhang[1], Chong Liu[1], Dong Liu[1,2,15,*]

[1] Ningbo Research Institute, State Key Laboratory of Modern Optical Instrumentation, College of Optical Science and Engineering, Zhejiang University, Hangzhou 310027, China;
[2] Intelligent Optics & Photonics Research Center, Jiaxing Key Laboratory of Photonic Sensing & Intelligent Imaging, Jiaxing Research Institute, Zhejiang University, Jiaxing 314000, China
[3] Univ. Littoral Côte d'Opale, CNRS, Univ. Lille, IRD, UMR 8187 - LOG - Laboratoire d'Océanologie et de Géosciences, F-62930 Wimereux, France
[4] Institute of Marine Sciences (ISMAR), Italian National Research Council (CNR), Rome - Tor Vergata, 00133, Italy
[5] Sorbonne Université, CNRS, LATMOS, 96 Boulevard de l'Observatoire, 06304 Nice Cedex, France
[6] Scuola di Ingegneria, Università della Basilicata, Viale Ateneo Lucano 10, I-85100 Potenza, Italy
[7] Cooperative Institute for Research in Environmental Sciences, University of Colorado Boulder and NOAA Chemical Sciences Laboratory, 325 Broadway, Boulder, Colorado 80305, USA
[8] Institute of Physics, National Academy of Sciences of Belarus, Pr. Nezavisimosti 68-2, Minsk 220072, Belarus
[9] Key Laboratory for Ecological Environment in Coastal Areas (State Oceanic Administration), National Marine Environmental Monitoring Center, Dalian 116023, China
[10] CAS Key Laboratory of Tropical Marine Bio-Resources and Ecology, South China Sea Institute of Oceanology, Chinese Academy of Sciences, Guangzhou 510301, China
[11] School of Atmospheric Sciences and Guangdong Province Key Laboratory for Climate Change and Natural Disaster Studies, Sun Yat-sen University, Zhuhai 519000, China
[12] Faculty of Technology and Environment, Prince of Songkla University, Phuket 83120, Thailand.
[13] Second Institute of Oceanography, Ministry of Natural Resources, Hangzhou 310012, China
[14] Ocean College, Zhejiang University, Zhoushan 316021, China
[15] Donghai Laboratory, Zhoushan 316021, China

† Equal contribution

Correspondence: *liudongopt@zju.edu.cn

Received: date; Accepted: date; Published: date

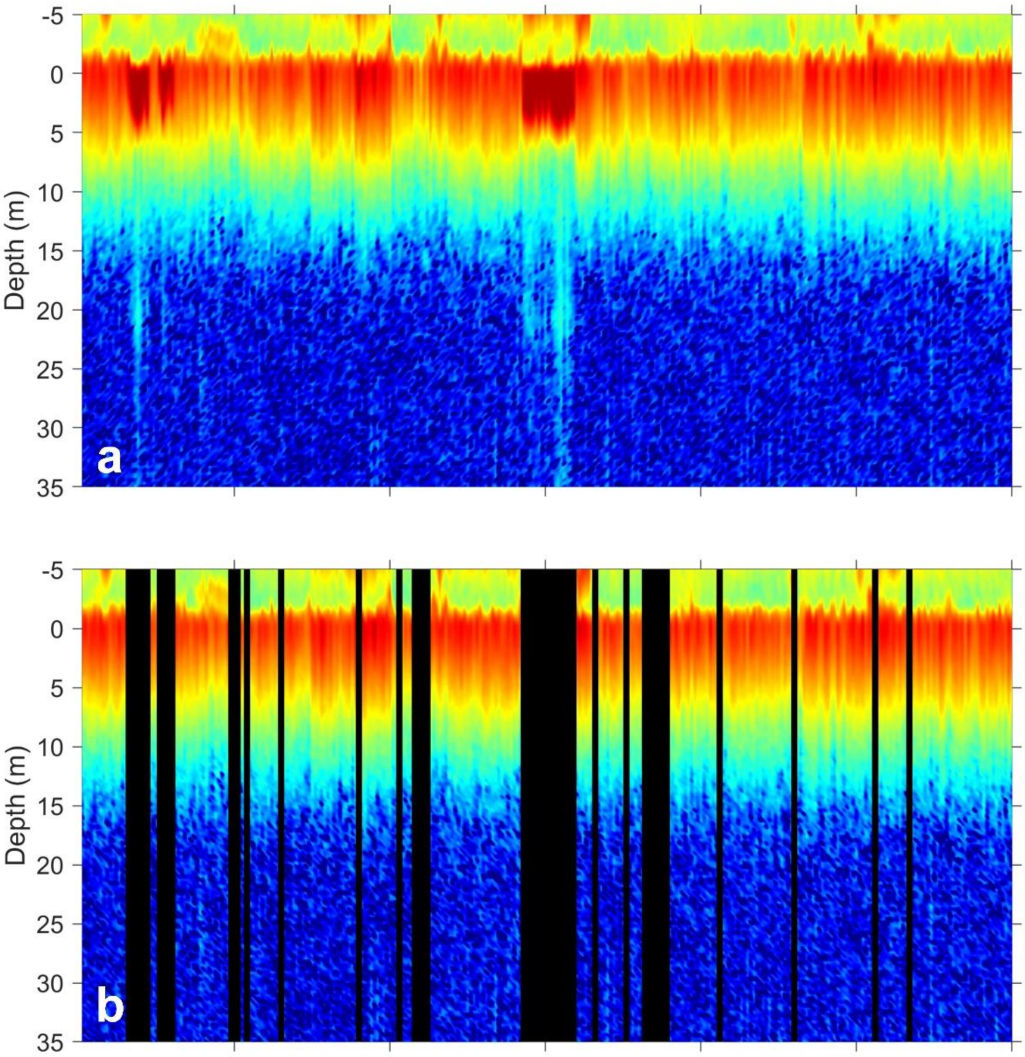

Fig. S1. Automatic removal of the sea foam. **a** Before, **b** after removal.

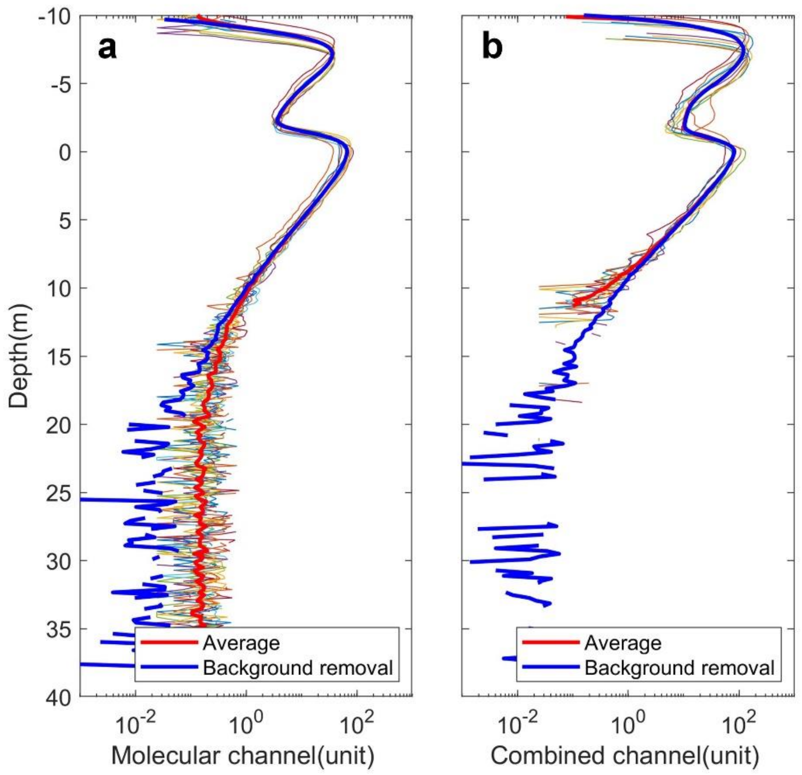

Fig. S2. Denoising algorithm to remove the background and random noises. **a** Molecular channel. **b** Combined channel.

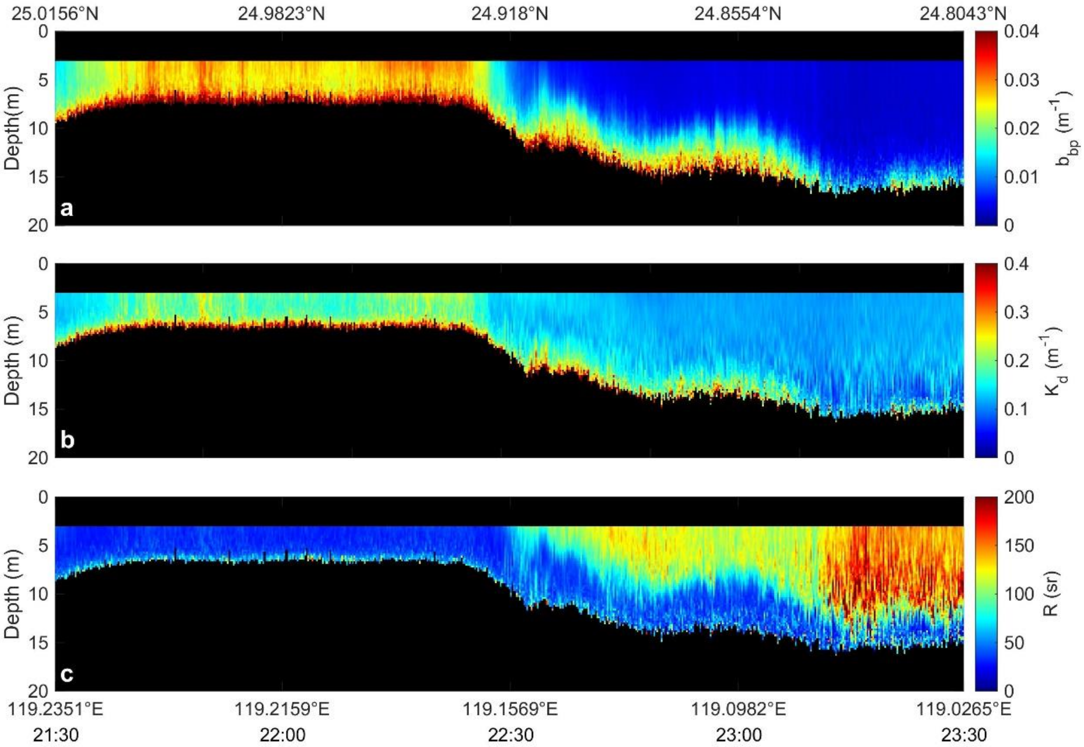

Fig. S3. The scattering layer observed by the HSRL around (24.8265° N, 119.0669° E). a Particulate backscattering coefficient. **b** Diffuse attenuation coefficient. **c** Lidar ratio.

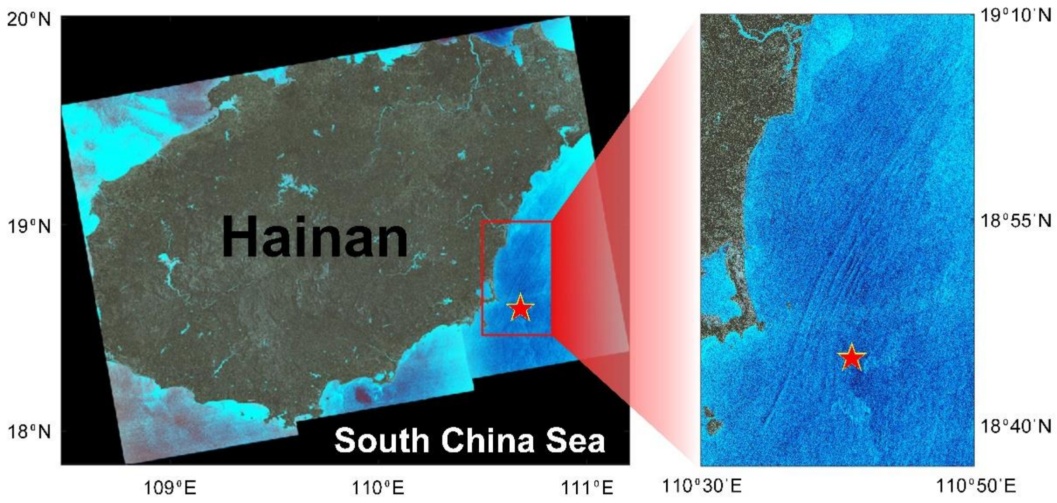

Fig. S4. Internal waves. Sentinel-1A Synthetic Aperture Radar quick-look image of internal waves in the Shimei Bay taken at the same day as the lidar observation (10:46:16 UTC on Sep. 14, 2020) [1] (copyright European Space Agency). Red star is the location of fixed station S_6.

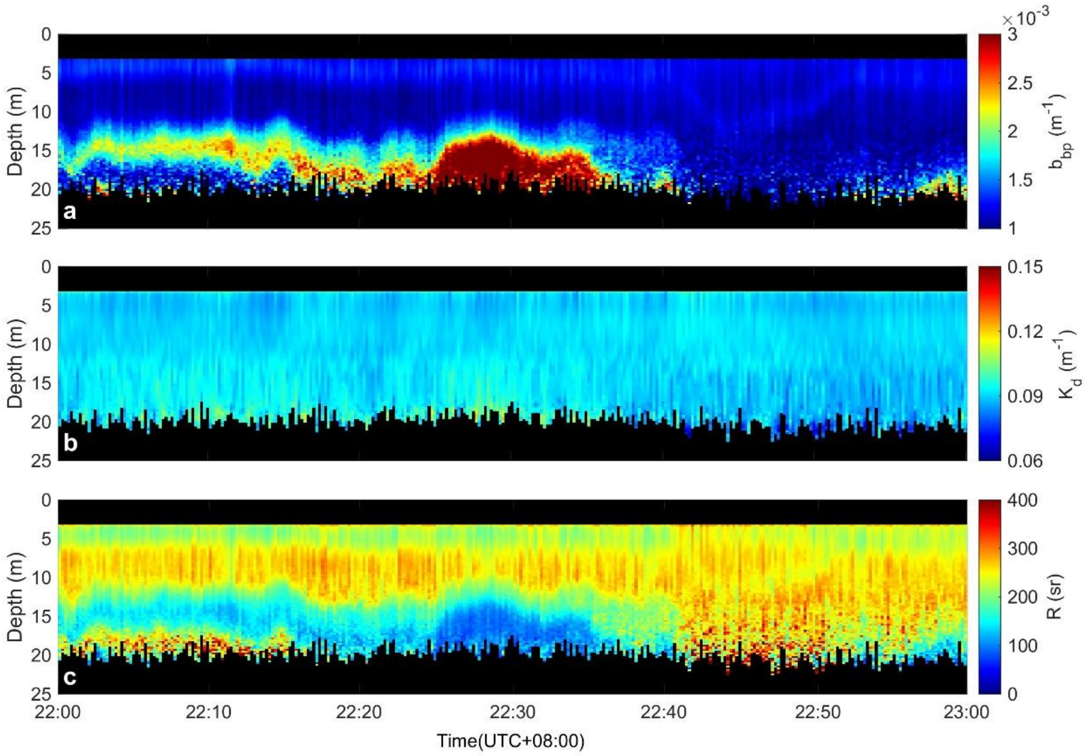

Fig. S5. The scattering layer observed by the HSRL around T5. a Particulate backscattering coefficient. **b** Diffuse attenuation coefficient. **c** Lidar ratio.

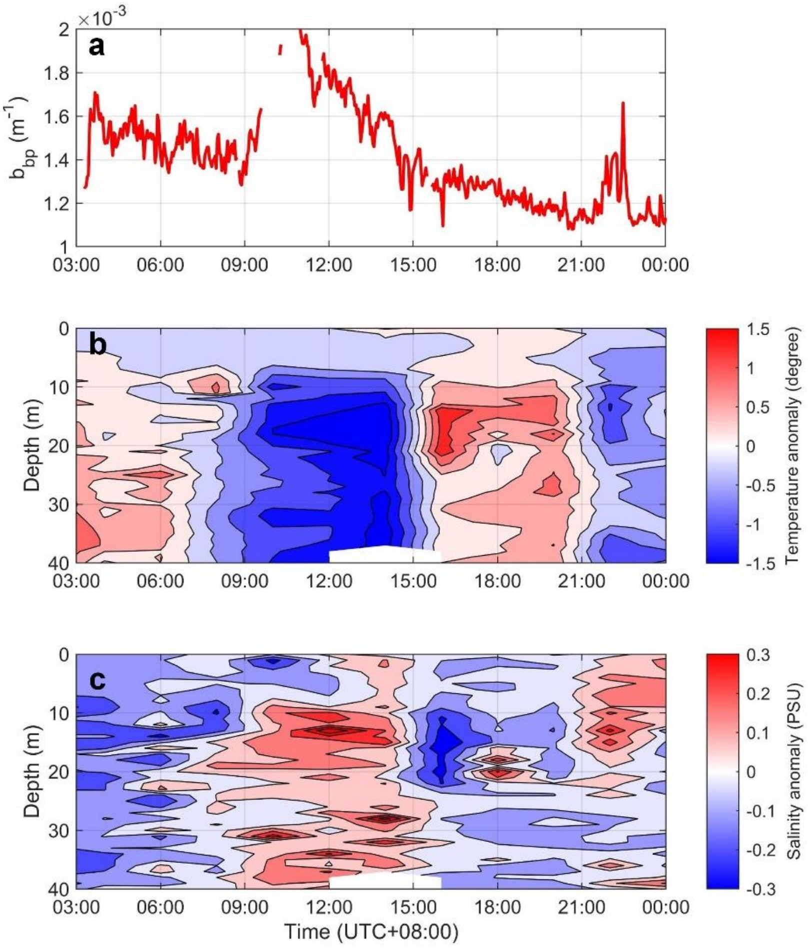

Fig. S6. a The average of particulate backscattering coefficient of HSRL within the depth of 5-15 m. Anomalies of **b** temperature, **c** salinity.

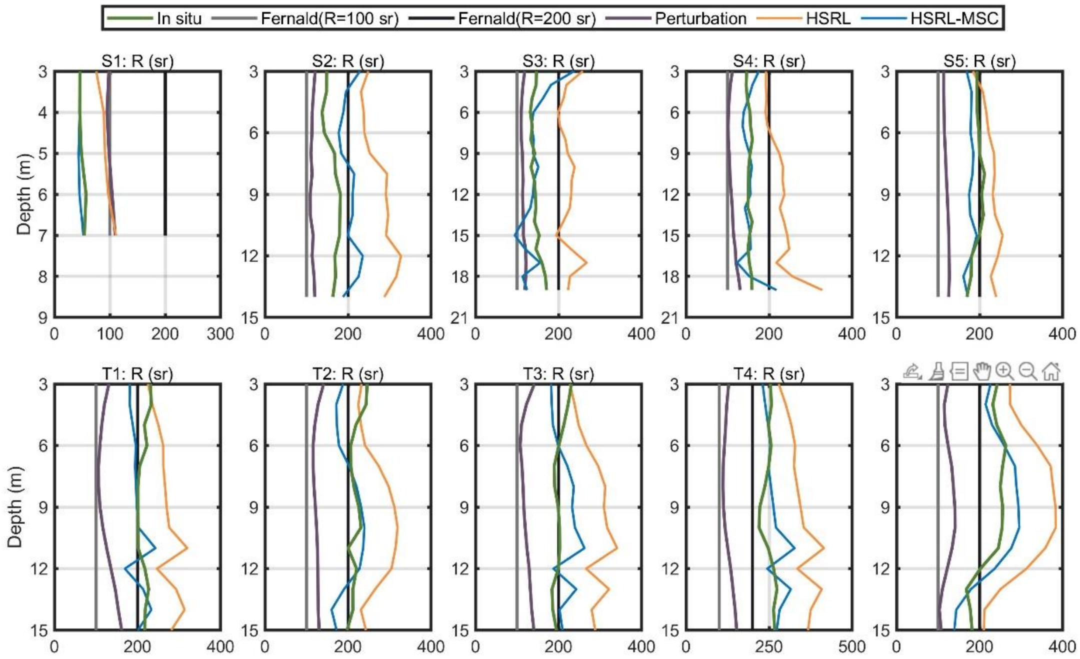

Fig. S7. The lidar ratio measured by the HSRL and *in situ* devices and assumed by the Fernald method and perturbation method.

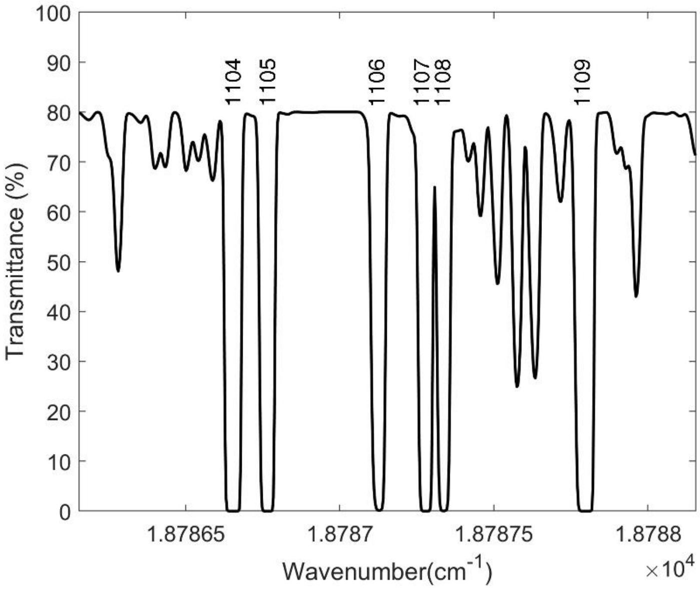

Fig. S8. Iodine absorption cell. a The picture of the packed iodine absorption cell integrated with the temperature controller. The light enters the device from the left and leaves from the right. **b** The absorption lines of iodine cell that might be employed in the HSRL.

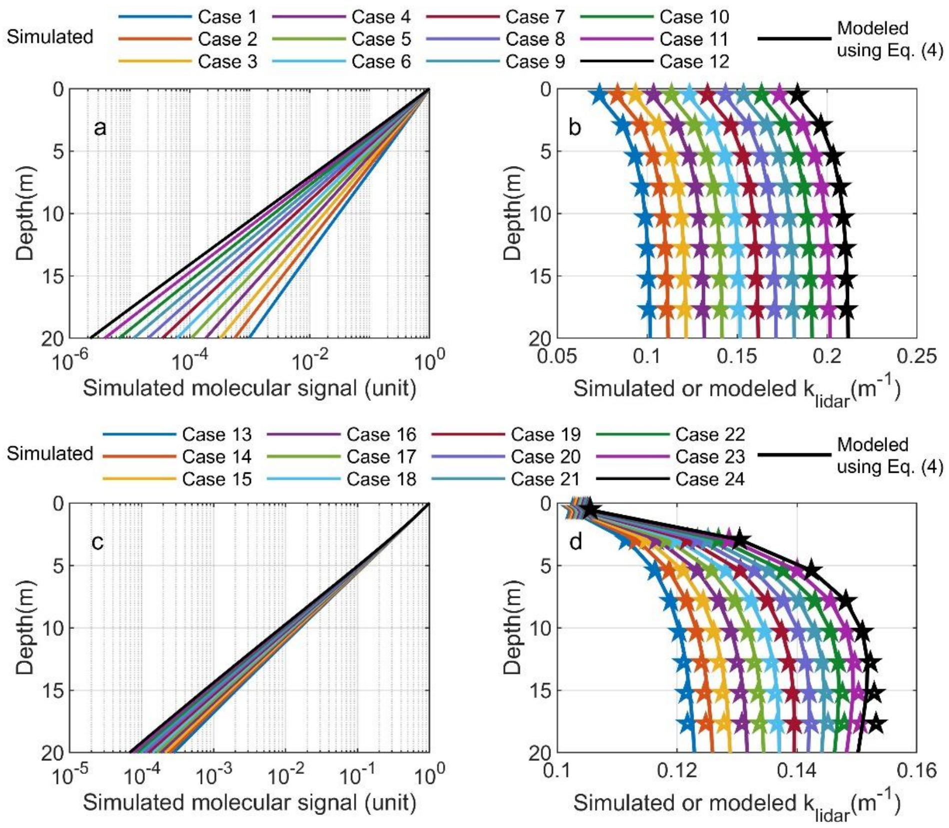

Fig. S9. Simulation under HSRL conditions in underway observation. **a** and **c** are molecular signals. **b** and **d** are k_{lidar} derived from Eq. (3). The simulation conditions for IOPs are listed in Table S2.

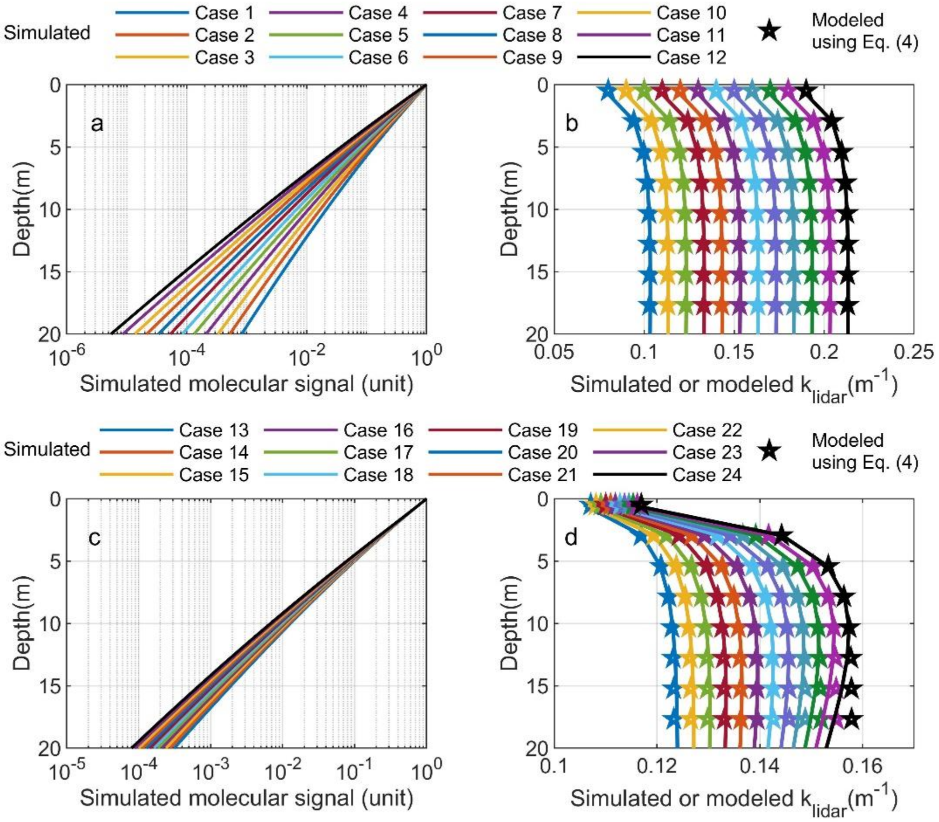

Fig. S10. Simulation under HSRL conditions at the fixed station. **a** and **c** are molecular signals. **b** and **d** are k_{lidar} derived from Eq. (3). The simulation conditions for IOPs are listed in Table S2.

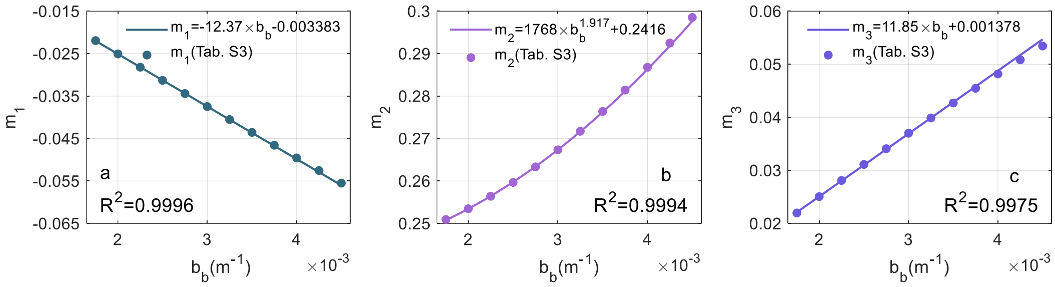

Fig. S11. Relationship between parameters **a** m_1, **b** m_2 and **c** m_3 and backscattering coefficient in underway measurement

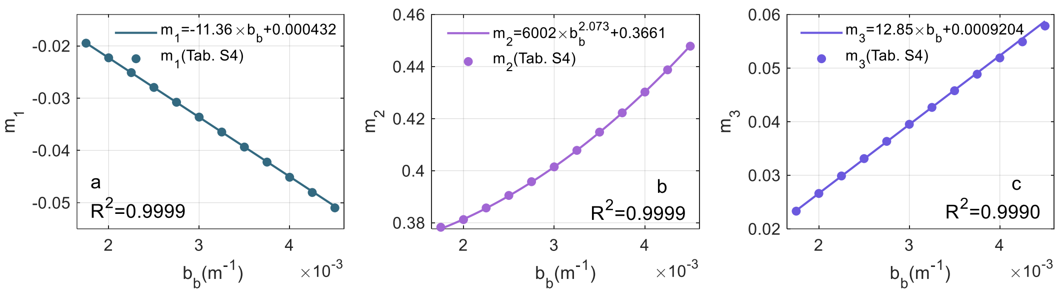

Fig. S12. Relationship between parameters **a** m_1, **b** m_2 and **c** m_3 and backscattering coefficient at the fixed station

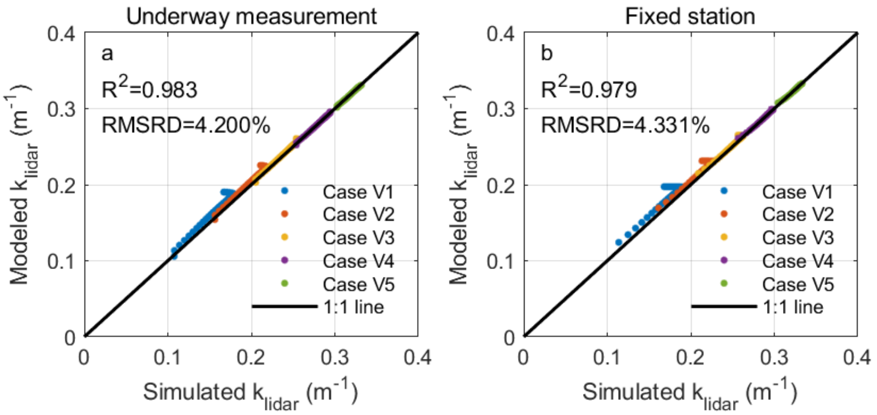

Fig. S13. Comparison between simulated and modeled k_{lidar}. **a** Underway measurement. **b** Fixed station. The simulation conditions for IOPs are listed in Table S4.

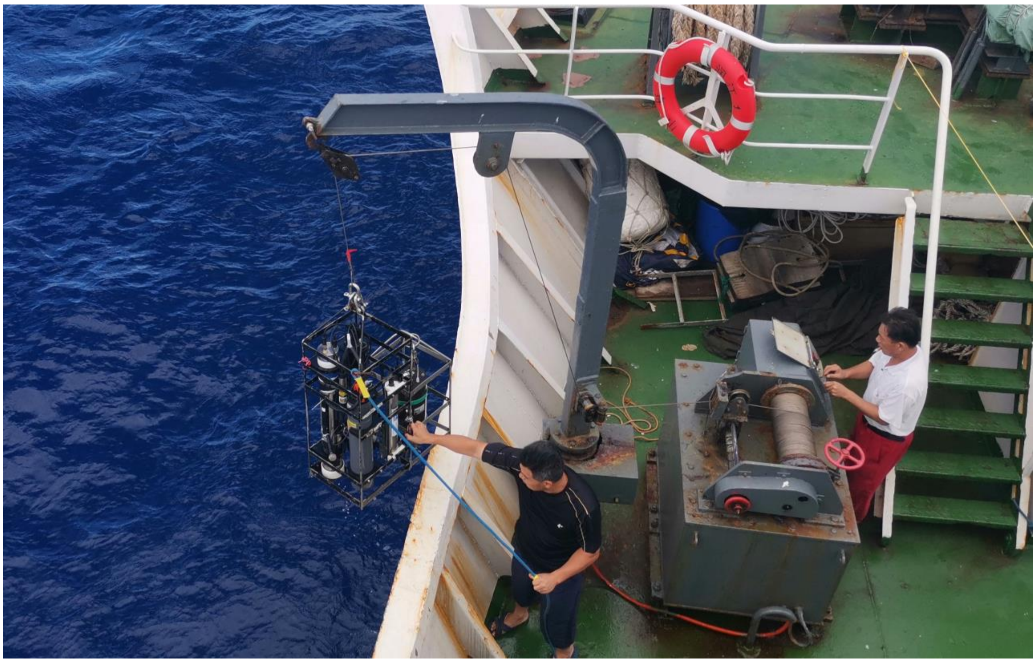

Fig. S14. *In situ* instruments were put into the seawater by a winch.

Tab. S1 Key parameters of oceanic HSRL

Parameter	Value	Unit
**Transmitter**		
Wavelength	532.2928	nm
Spectral bandwidth	75	MHz
Spectral stability	15	MHz
Pulse energy	10	mJ
Pulse width	10	ns
Pulse repetition rate	10	Hz
Beam divergence	1	mrad
**Receiver**		
Aperture diameter	50.8	mm
Field of view	200	mrad
Optical filter bandwidth	3	nm
Iodine cell absorption line	1104	-
Sample rate	400	MSa/s
Electronic bandwidth	100	MHz

Tab. S2 IOPs in simulation of molecular signals for establishing model

Case	absorption coefficient a (m^{-1})	backscattering coefficient b (m^{-1})	backscattering coefficient b_b (m^{-1})
**1**	0.07	0.10	0.0025
**2**	0.08	0.10	0.0025
**3**	0.09	0.10	0.0025
**4**	0.10	0.10	0.0025
**5**	0.11	0.10	0.0025
**6**	0.12	0.10	0.0025
**7**	0.13	0.10	0.0025
**8**	0.14	0.10	0.0025
**9**	0.15	0.10	0.0025
**10**	0.16	0.10	0.0025
**11**	0.17	0.10	0.0025
**12**	0.18	0.10	0.0025
**13**	0.10	0.07	0.00175
**14**	0.10	0.08	0.002
**15**	0.10	0.09	0.00225
**16**	0.10	0.10	0.0025
**17**	0.10	0.11	0.00275
**18**	0.10	0.12	0.003
**19**	0.10	0.13	0.00325
**20**	0.10	0.14	0.0035
**21**	0.10	0.15	0.00375
**22**	0.10	0.16	0.004
**23**	0.10	0.17	0.00425
**24**	0.10	0.18	0.0045

Tab. S3 m_1, m_2, m_3 and R^2 under HSRL conditions in underway observation

Case	m_1	m_2	m_3	R^2
**1**	-0.0313	0.2597	0.0311	0.997
**2**	-0.0313	0.2597	0.0311	0.997
**3**	-0.0313	0.2597	0.0311	0.997
**4**	-0.0313	0.2597	0.0311	0.997
**5**	-0.0313	0.2597	0.0311	0.997
**6**	-0.0313	0.2597	0.0311	0.997
**7**	-0.0313	0.2597	0.0311	0.997
**8**	-0.0313	0.2597	0.0311	0.997
**9**	-0.0313	0.2597	0.0311	0.997
**10**	-0.0313	0.2597	0.0311	0.997
**11**	-0.0313	0.2597	0.0311	0.9997
**12**	-0.0313	0.2597	0.0311	0.997
**13**	-0.0219	0.2509	0.0220	0.992
**14**	-0.0251	0.2535	0.0251	0.994
**15**	-0.0282	0.2564	0.0281	0.996
**16**	-0.0313	0.2597	0.0311	0.997
**17**	-0.0344	0.2633	0.0341	0.998
**18**	-0.0375	0.2673	0.0370	0.999
**19**	-0.0405	0.2717	0.0399	0.999
**20**	-0.0436	0.2764	0.0427	0.999
**21**	-0.0466	0.2814	0.0455	0.998
**22**	-0.0496	0.2868	0.0482	0.996
**23**	-0.0525	0.2925	0.0508	0.993
**24**	-0.0555	0.2985	0.0534	0.990

Tab. S4 m_1, m_2, m_3 and R^2 under HSRL conditions at fixed station

Case	m_1	m_2	m_3	R^2
**1**	-0.0279	0.3905	0.0331	0.994
**2**	-0.0279	0.3905	0.0331	0.994
**3**	-0.0279	0.3905	0.0331	0.994
**4**	-0.0279	0.3905	0.0331	0.994
**5**	-0.0279	0.3905	0.0331	0.994
**6**	-0.0279	0.3905	0.0331	0.994
**7**	-0.0279	0.3905	0.0331	0.994
**8**	-0.0279	0.3905	0.0331	0.994
**9**	-0.0279	0.3905	0.0331	0.994
**10**	-0.0279	0.3905	0.0331	0.994
**11**	-0.0279	0.3905	0.0331	0.994
**12**	-0.0279	0.3905	0.0331	0.994
**13**	-0.0195	0.3783	0.0233	0.988
**14**	-0.0223	0.3812	0.0266	0.990
**15**	-0.0251	0.3857	0.0299	0.992
**16**	-0.0279	0.3905	0.0331	0.994
**17**	-0.0308	0.3958	0.0363	0.995
**18**	-0.0336	0.4015	0.0395	0.995
**19**	-0.0365	0.4079	0.0427	0.994
**20**	-0.0394	0.4148	0.0458	0.992
**21**	-0.0422	0.4222	0.0489	0.988
**22**	-0.0451	0.4302	0.0519	0.984
**23**	-0.0480	0.4387	0.0549	0.976
**24**	-0.0510	0.4478	0.0579	0.966

Tab. S5 IOPs in simulation of molecular signals for validating model

Case	absorption coefficient a (m^{-1})	backscattering coefficient b (m^{-1})	backscattering coefficient b_b (m^{-1})
**V1**	0.1	0.3	0.0075
**V2**	0.15	0.25	0.00625
**V3**	0.2	0.2	0.005
**V4**	0.25	0.15	0.00375
**V5**	0.3	0.1	0.0025

Section S1:

Lidar signals are dependent on the backscatter and attenuation. Therefore, it is an ill-posed problem to retrieve two properties from one equation. Fernald method was initially developed in the atmospheric lidar [2] with the assumption of the lidar backscatter to extinction ratio. Recently, Fernald method was employed into the lidar for detection of water optical properties [3]. Theoretical and experimental results show that the lidar attenuation coefficient is close to diffuse attenuation coefficient K_d when the field of view of the HSRL is large. Then, K_d can be calculated from the signal B_C of the combined channel of HSRL as

$$\begin{aligned} K_{\mathrm{d}}(z) &= K_{\mathrm{d,p}}(z) + K_{\mathrm{d,w}}(z) \\ &= (1-R_S)K_{\mathrm{d,w}}(z) + \frac{B_{\mathrm{C}}(z)\Phi(z)}{\dfrac{B_{\mathrm{C}}(z_c)}{K_{\mathrm{d,p}}(z_c)+R_S K_{\mathrm{d,w}}(z_c)} + 2\int_z^{z_c} B_{\mathrm{C}}(z)\Phi(z)\mathrm{d}z} \end{aligned} \quad \text{(S1)}$$

where z is the depth, $K_{\mathrm{d,p}}$ and $K_{\mathrm{d,w}}$ are the diffuse attenuation coefficient of the suspended matter and pure seawater, respectively, R_S is the ratio between the lidar ratios of the suspended matter R and water molecules R_w, $\Phi(z) = \exp\left[2(R_S - 1)\int_z^{z_c} K_{\mathrm{d,m}}(z)\mathrm{d}z\right]$, z_c is the boundary depth and $K_{\mathrm{d,p}}(z_c)$ can be estimated by the slope method.

Section S2:

In situ instruments were put into the seawater by a winch to collect inherent optical properties, as shown in Fig. S14. WETLabs acs was used to collect the absorption coefficient and beam attenuation coefficient at 532 nm. The pure water absorption and attenuation were corrected considering the changes due to the temperature and salinity. The coincident temperature, salinity, and depth data were provided by a Sea-Bird Electronics, Inc. (SBE) conductivity-temperature-depth (CTD). The scattering errors in the particulate absorption measurement were corrected [4]. The backscattering coefficient at 510 nm was measured by HOBILabs HS6P using the scattering at a given angle of ~140 degrees. Sigma correction was done according to the HOBILabs operation manual [5]. Because of the absence of 532 nm data of HS6P, the backscattering at 532 nm was estimated by that at 510 nm through a factor of (532/510). The transfer error is limited because 532 nm is very close to 510 nm. The *in situ* K_d was calculated according to the algorithm by Lee [6] from the *in situ* absorption and backscattering coefficients. Through the quality control, all *in situ* data were then binned to depth resolution of 1 m.

Reference:
1. Copernicus Sentinel data [2021]. Retrieved from ASF DAAC, processed by ESA.
2. Fernald, F.G. Analysis of atmospheric LIDAR observations : Some comments. *Applied Optics* **23**, 652-653 (1984).
3. Zhou, Y. et al. Detecting atmospheric-water optical properties profiles with a polarized lidar. *National Remote Sensing Bulletin* **23**, 108-115 (2019).
4. Zaneveld, J.R.V., Kitchen, J.C. & Moore, C.C. Scattering error correction of reflecting-tube absorption meters. Ocean Optics XII: International Society for Optics and Photonics. 44-56 (1994).
5. HOBILabs. HydroScat-6P User's Manual: Revision J. 2010.
6. Lee, Z.P., Du, K.P. & Arnone, R. A model for the diffuse attenuation coefficient of downwelling irradiance. *J. Geophys. Res. Oceans* **110**, 93-106 (2005).
